# Diagnostic value of FDG-PET/CT in fever of unknown origin

**DOI:** 10.1186/s43055-022-00725-z

**Published:** 2022-03-01

**Authors:** Maha Omar Mohamed Elshalakani, Nivine Chalabi, Hanan Mohamed Hanafy, Amal Ibrahim Ahmed Othman

**Affiliations:** Radiology Department, Faculty of Medicine, Ain Shams University, Cairo, 11566 Egypt

**Keywords:** FDG PET/CT, Fever of unknown origin

## Abstract

**Background:**

Fever of unknown origin (FUO) is a challenging clinical problem in medicine that needs collaboration of various diagnostic techniques to establish the accurate diagnosis. We evaluated the diagnostic performance of 18F-FDG PET/CT in patients who presented themselves with FUO. Our study included 40 patients with FUO who underwent PET/CT examination and their results were compared to the results of laboratory, histopathological, microbiological investigations and/or response to therapy.

**Results:**

The final diagnosis included malignancy in 20 patients (50%), infectious causes in 7 patients (17.5%) and non-infectious inflammatory causes in 6 patients (15%). Fever resolved without diagnosis in 4 patients (10%), while no definite diagnosis was reached in 3 patients (7%). PET/CT successfully contributed to diagnosis of 35 out of 40 patients with diagnostic accuracy of 87.5%. The sensitivity, specificity, positive predictive value and negative predictive value of PET/CT in our study were 93.5%, 66.7%, 90.6% and 75%, respectively.

**Conclusion:**

PET/CT is a useful tool to investigate and diagnose the cause of FUO. It provides information that can guide the treatment strategy of the patients.

## Background

Fever of unknown origin is defined as a continuous or intermittent fever equal to or above 38.3 centigrade, which is there for more than 3 weeks without an obvious cause along 1 week of extensive investigations during hospitalization. Immunocompromised patients are not included [1, 2] according to Petersdorf and Beeson [3] who first put the definition of fever of unknown origin.

FUO can be caused by a wide range of underlying disorders, including infections, occult malignancies, and non-infectious inflammatory diseases (NIID). These diseases are highly heterogeneous in nature, and are managed by several different medical specialties, which further complicates early diagnosis [4].

Infections were the most diagnosed cause for classical FUO in most published studies, accounting for approximately one third of all cases [5]. Another third of FUO cases is due to non-infectious inflammatory disorders (NIID) such as adult Still disease, giant cell/temporal arteritis, and systemic lupus erythematosus (SLE) [6]. Neoplasms and malignancies account for up to 18% of FUO etiologies. The most common neoplasms associated with FUO are lymphoma and renal cell carcinoma, followed by acute myeloid leukemia and myeloproliferative disorders [6].

Positron emission tomography/computerized tomography (PET/CT) is a functional image modality using its ability to metabolize glucose and concentrate specific molecules that have been labelled with a positron-emitting radionuclide. This unique characteristic of 18F-FDG PET/CT may be used for differential diagnosis of fever of unknown origin (FUO) [7].

Although the first combined PET/computed tomography (CT) system became operational in 2001 already, only few studies have assessed 18F-FDG PET/CT in patients with FUO. Furthermore, it is not clear when to perform an 18F-FDG PET/CT for diagnostic work-up of FUO [8].

In this study, we aim to evaluate the potential clinical contribution of 18-fluoro-2-deoxyglucose positron emission tomography/computed tomography (18F-FDG-PET/CT) in the identification of the underlying cause of FUO and to compare the results with the final diagnosis of the patients according to histopathological, microbiological, other laboratory investigations and/or response to therapy.

## Methods

### Study population

This study included 40 patients with fever of unknown etiology who were referred for PET/CT assessment from July 2019 to September 2021. This prospective study was carried out after the approval of The Ethical Committee of Scientific Research, Faculty of Medicine, Ain Shams University. All participants in this study provided informed consent and confidentiality was ensured.

The inclusion criteria included the diagnostic criteria of fever of unknown origin (FUO) which are: Temperature of at least 38.3 °C on at least two occasions, duration of illness of at least 3 weeks or multiple febrile episodes in at least 3 weeks, not immunocompromised and diagnosis is uncertain despite thorough assessment of history, physical examination, and investigations.

Exclusion criteria included: patients with contraindications to PET CT, e.g.: high blood glucose level, patients with history of atopic disorders and patients with renal function impairment (with serum creatinine > 1.5 mg/dl) and patients with bad general condition needing life support.

### PET/CT examination

PET/CT study was performed using a dedicated hybrid PET/CT scanner GE medical system [(GE discovery IQ 5 rings) and enhanced helical CT (optima 540 16-slice)]. Instructions were given to the patients to avoid strenuous exercise for several days and to fast from all types of food and drinks except water for a minimum of 4–6 h prior to the examination. Diabetic patients were advised to eat breakfast and take their hypoglycemic medications for at least 6 h before the examination. Serum glucose levels were measured to ensure euglycemia (Fasting blood glucose level < 200 mg/dl) before the tracer injection. Patients were asked to empty their urinary bladder before the study.

### Imaging technique

While resting on a reclining chair, approximately 0.06–0.08 mci/kg of 18F-FDG were injected intravenously with saline infusion followed by 45–60 min of rest then all patients were positioned on the imaging tables with their arms up. Approximately1-2 ml/kg of iodinated non-ionic low osmolar contrast medium was injected intravenously for the contrast enhanced CT scans. Contrast enhanced CT scanning were initiated at the orbito-meatal line and progressed to the mid-thigh (28–30 mAs; 120 kV; slice thickness 5 mm); the corresponding PET imaging immediately followed over the same body region. It was divided into many positions depending on the extent of the study, starting the examination distance from the skull base to mid-thigh level including 6–7 positions with 3 min acquisition time for each position. Patients were instructed to breathe shallowly during the PET and CT portions of the study to minimize mis-registration between the PET and CT images. The CT data were used for attenuation correction, and the images were reconstructed. Delayed PET CT images after 120 min were needed in some cases when the uptake of the lesion was controversial.

### Data interpretation

Image data were interpreted by at least two nuclear medicine radiologists using a work station with fusion software (GE healthcare), which provides multi-planar reformatted images and displayed PET images, CT images, and PET/CT fusion images. The study was considered positive when at least one site of pathological FDG uptake was encountered. It was defined as negative study when only physiological FDG biodistribution could be identified. PET/CT was then categorized into contributory and non-contributory to the final diagnosis.

### PET/CT was considered contributory to diagnosis in the following conditions

PET/CT study was considered contributory to diagnosis when the study was either true positive or true negative. When PET/CT study showed focal pathological uptake that was confirmed by additional diagnostic test or clinical evaluation to be the source of FUO, it was considered true positive (TP) study. It was defined as true negative (TN) when only physiological distribution of FDG was found and no other diagnosis could be reached by conventional diagnostic procedures. Therefore, the patient was discharged from hospital either fever free or with fever of unknown origin as the final diagnosis.

### PET/CT was considered non-contributory to diagnosis in the following conditions:

When PET/CT study showed focal abnormal FDG uptake that was reported as suspicious for malignancy and led to unnecessary further tissue biopsy to confirm the diagnosis, it was considered false positive (FP) study. Also, it was considered false positive when an alternative diagnosis was confirmed by other investigations.

A false negative (FN) study is that with normal PET/CT, yet the diagnosis of the patient was confirmed by other investigations.

### Final diagnosis

Final diagnosis of the patient was defined according to further diagnostic tests and clinical evaluation that followed PET/CT examination to confirm/rule out the diagnosis. All cases with suggested malignancy as an etiology of the FUO underwent tissue diagnosis guided by the pathological sites suggested in PET/CT. The other categories of diagnosis were confirmed by detailed clinical, laboratory, sometimes pathological tests and/or follow-up according to each category. The final diagnoses were categorized into: malignant, infectious, non-infectious inflammatory disease (NIID), other (resolution of fever with no diagnosis reached) and no diagnosis reached with persistence of fever.

### Statistical analysis

Analysis of data was done using IBM SPSS (Statistical package for social science) program version 22. To describe the studied sample, quantitative data were presented as mean, median and standard deviations. Qualitative data were presented as count & percentage. The Chi square statistic is used for testing relationships between categorical variables. Sensitivity, specificity, positive predictive value, negative predictive value and accuracy were calculated.

## Results

### Demographic data

Out of the 40 patients participated in the study, 28 were males (70%) and 12 were females (30%) with age ranging from 8 to 80 years (mean age was 41.25 years with SD of 19.6). Five patients were aged less than 16 years old.

### Final Diagnosis of the patients

**Fig. 1:**
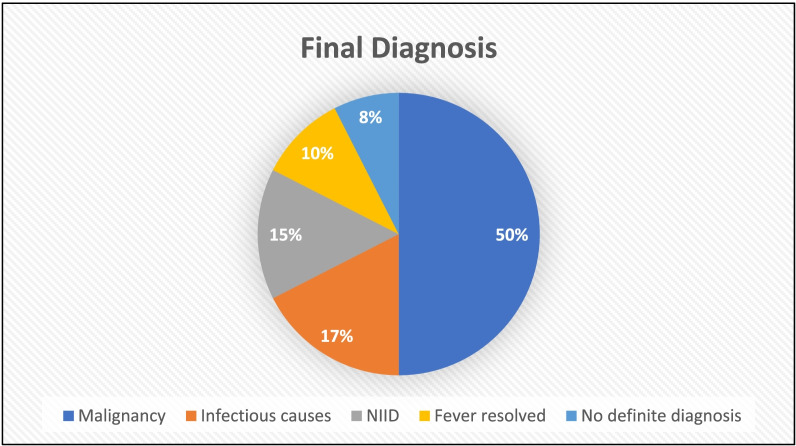
Final diagnosis of patients included in the study

The final diagnosis included malignancy in 20 patients (50%), infectious causes in 7 patients (17.5%) and non-infectious inflammatory causes (NIID) in 6 patients (15%). Fever resolved without diagnosis in 4 patients (10%), while no definite diagnosis was reached in 3 patients (7%) (Fig. 1).

### Malignant causes

**Fig. 2:**
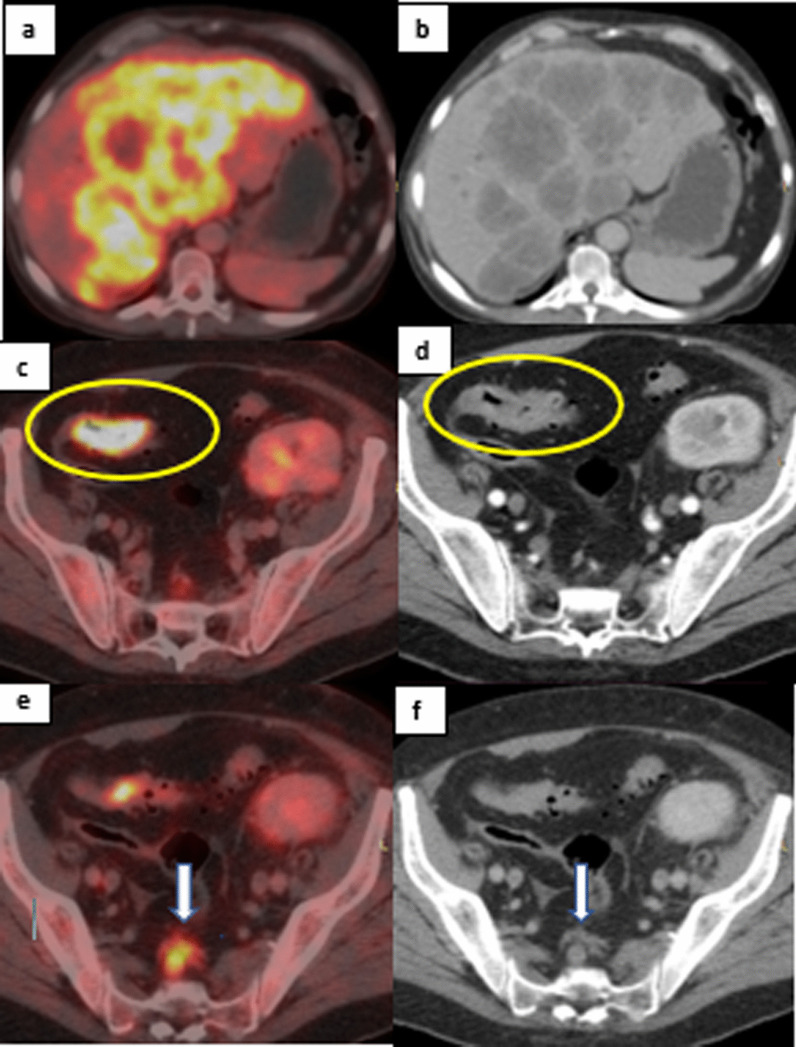
62-year-old male presented with fever of unknown origin with history of renal transplantation. **a**, **b** Axial fused PET/CT image of the abdomen and same level axial CT of the abdomen showing large metabolically active hepatic focal lesions with central necrosis. **c**, **d** Axial fused PET/CT image and axial CT image of the lower abdomen showing small segment of sigmoid colon FDG avid mural thickening (yellow circles) that showed persistent increased activity in delayed imaging (not shown here). **e**, **f** Axial fused PET/CT and CT images of the upper pelvis showing regional enlarged metabolically active presacral lymph nodes (white arrows). Pathology from the sigmoid colon revealed adenocarcinoma

The whole 20 patients with malignant etiology as the final diagnosis underwent histopathological examination to confirm the diagnosis. Malignancies included Lymphoma in 15/20 (75%) (one had primary osseous lymphoma), osseous plasmacytoma in 1/20 (5%) Renal cell carcinoma in 1/20 (5%), Metastatic colonic cancer to the liver in 1/20 (5%) (Fig. 2), Lung cancer in 1/20 (5%) and pancreatic cancer with liver metastases in 1/20 (5%).

### Infectious causes

**Fig. 3:**
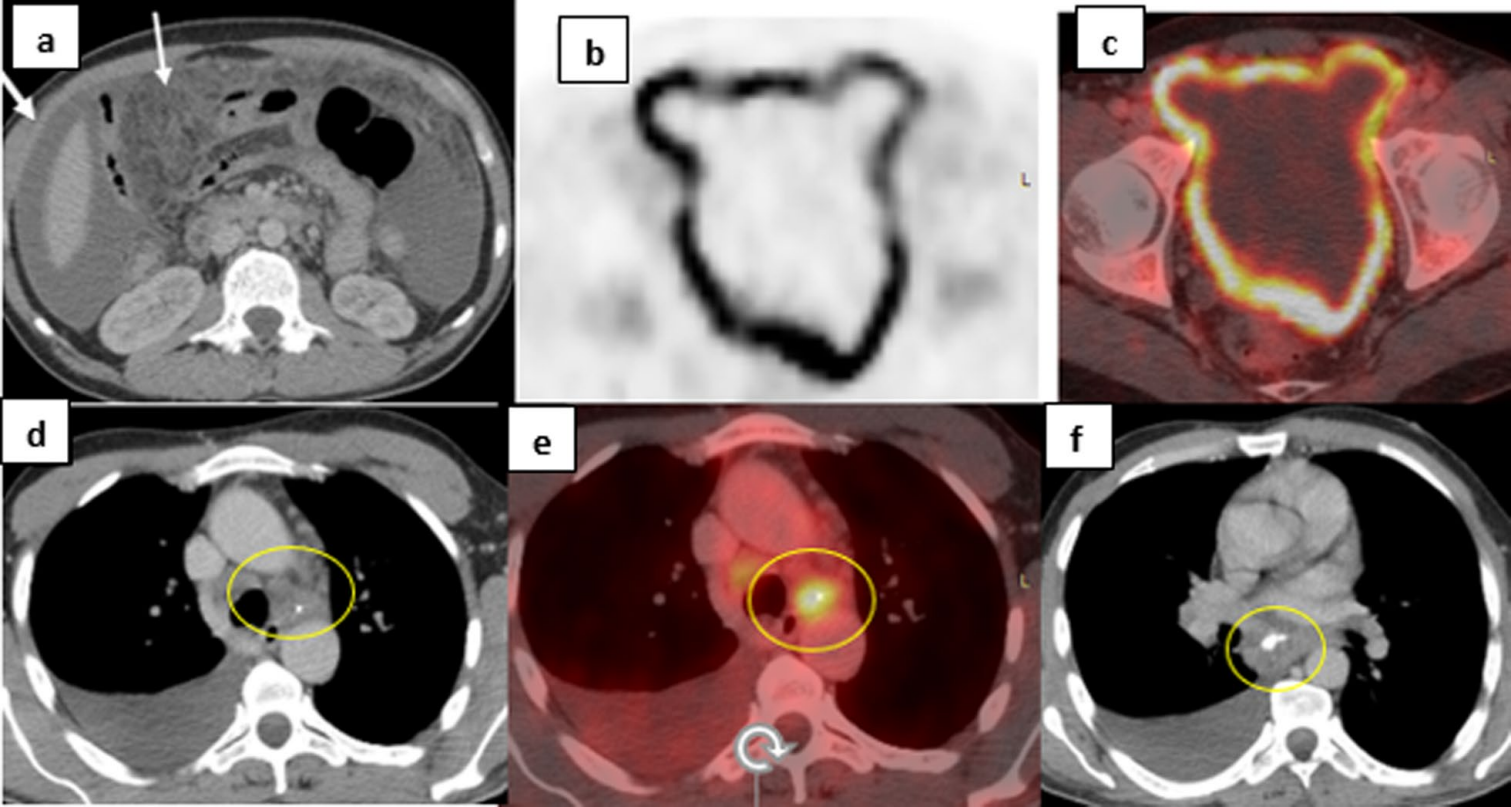
44-year-old male presented with fever of unknown etiology and ascites. **a** Axial CT of the abdomen showing diffuse peritoneal fat smudging with diffuse parietal peritoneal thickening (white arrows). **b**, **c** Axial PET and fused PET/CT axial image of the lower abdominal region showing diffuse hypermetabolic peritoneal thickening with SUVmax of 6 and moderate ascites. **d**, **f** Axial chest CT mediastinal window showing enlarged mediastinal lymph nodes with calcifications (yellow circles) and right sided pleural effusion. **e** Axial fused PET/CT image of those lymph nodes showing increased metabolic activity. Ascetic fluid sample stained positive for mycobacterium T.B. The patient improved on anti-tuberculous therapy

**Fig. 4:**
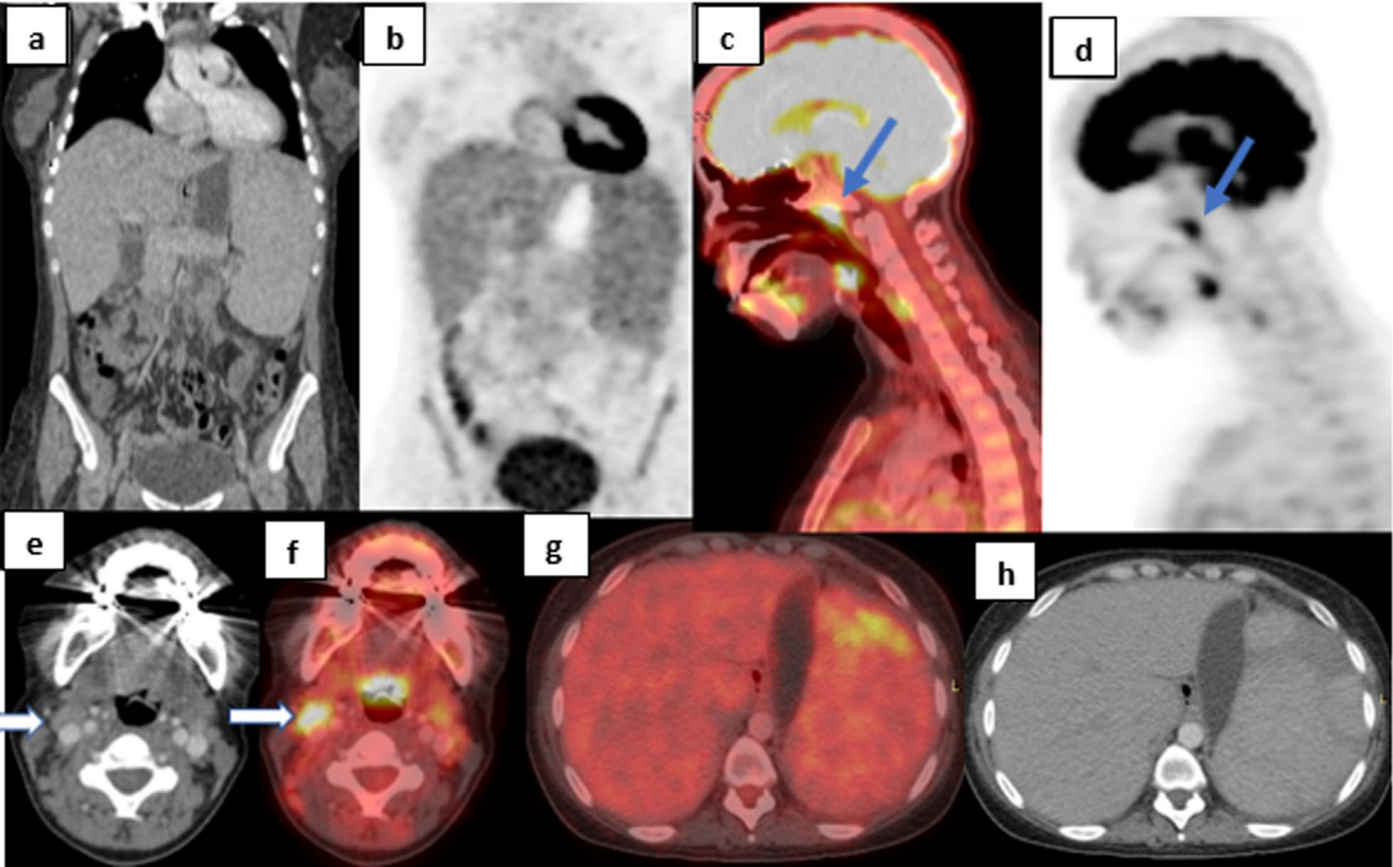
34-year-old female patient presented with fever of unknown etiology, fatigue and mild sore throat. **a** Coronal CT image, **b** Coronal PET image with attenuation correction showing enlarged liver and spleen with mildly increased splenic activity. **c** Sagittal PET/CT fused reconstructed image. **d** Sagittal PET attenuation corrected image, both showing increased nasopharyngeal activity with SUV max of (blue arrows). **e** Axial CT image of the neck. **f** Axial fused PET/CT image of the neck at the same level sowing right cervical metabolically active lymph nodes. **g**, **h** axial fused PET/CT and axial CT of the abdomen showing the enlarged spleen with ill-defined area of increased activity of its anterior pole. Other findings included diffuse increased marrow activity with SUV max of 3.3(not shown here). Her work-up showed positive Epstein Barr IgM

Infectious causes included TB in 2/7 patients (28.5%) (Fig. 3), infectious mononucleosis in 2/7 patients (28.5%) (Fig. 4), Urinary tract infection (UTI) in 2/7 (28.5%) and COVID-associated pneumonia in 1/7 patient (14%).

### Non-infectious inflammatory causes

**Fig. 5:**
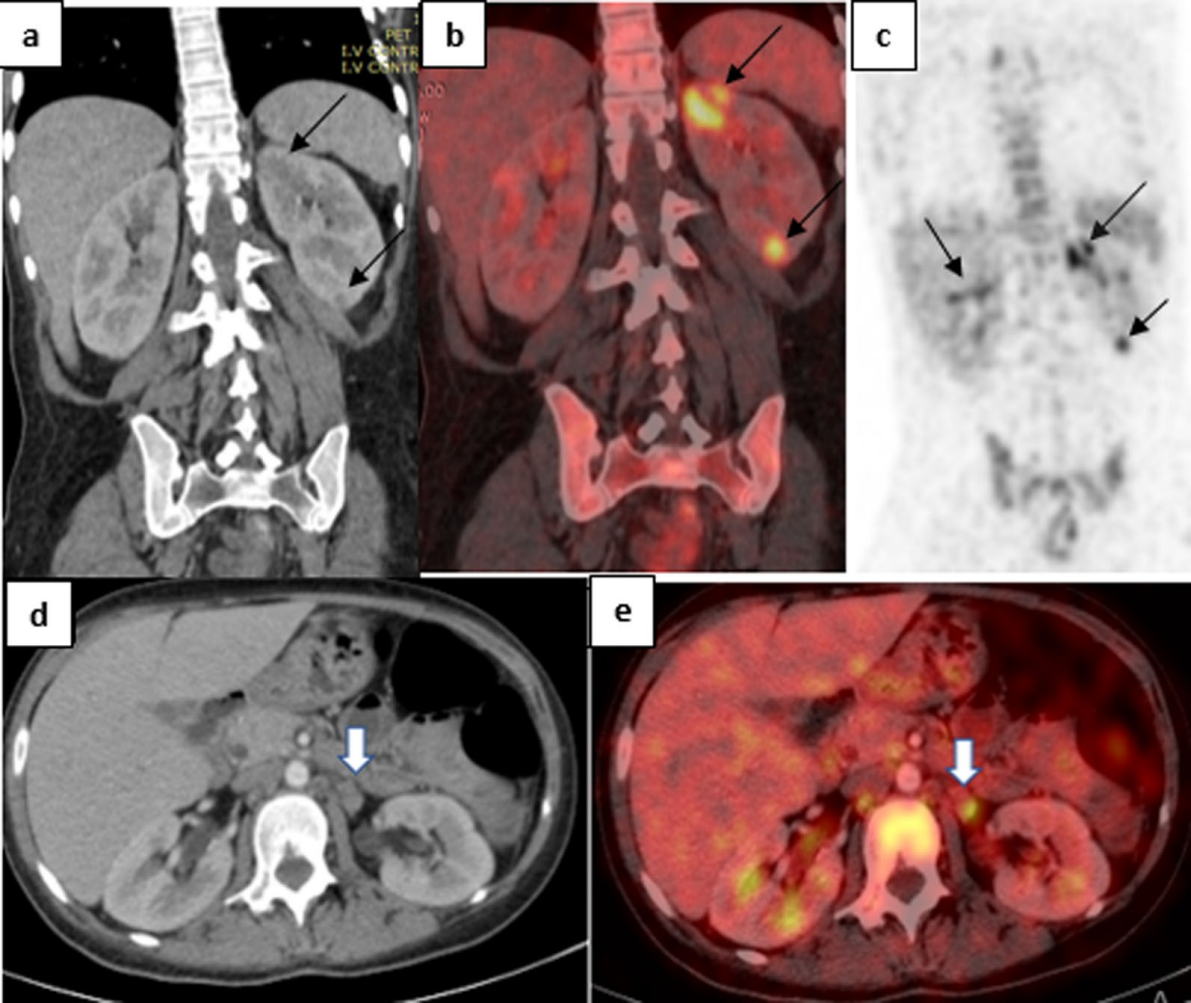
36-year-old female with history of SLE (systemic lupus erythromatosis) presented with fever of unknown origin. **a** Coronal CT image. **b** Coronal Fused PET/CT image and **c** Coronal PET image of the upper abdomen showing bilateral renal cortical hypodense metabolically active lesions (larger, more in number and more avid on the left side) (black arrows). **d**, **e** are axial CT and Fused PET/CT of the abdomen at the same level showing enlarged low metabolically active regional lymph nodes. The patient was diagnosed as lupus nephritis and responded to adequate treatment with appropriate doses of steroids

Non-infectious inflammatory causes included medium vessel vasculitis in 1/6 (16.6%), Sarcoidosis in 1/6 (16.6%), Familial Mediterranean Fever in 1/6 (16.6%), Still disease in 1/6 (16.6%), Lupus nephritis in 1/6 (16.6%) (Fig. 5) and Dermatomyositis in 1/6 (16.6%) of the patients.

SUV ranges in different categories of final diagnosis is illustrated in Table 1.

**Table 1:** Minimum and maximum SUV_max_ in different etiologies of FUO

Etiology	Minimum SUV_max_	Maximum SUV_max_	Mean SUV_max_ with margins of error (with 95% confidence interval)	Standard deviation
Malignant	6	45	12.92 ± 3.76	8.36
Infectious	3	8	4.8 ± 1.533	1.749
NIID	3.7	7.9	6.166 ± 1.164	1.45

### True positive cases (TP)

29 True positive cases (72.5%) were identified which included the whole malignant category (20/20) patients (100% of the malignant patients), four out of seven cases with infectious etiology (57.1%), and five out of six patients with Non-infectious inflammatory condition as an etiology (83.3%).

### True negative cases (TN)

Six studies were true negatives (15%). Fever resolved spontaneously in 3 patients, while in the other three, no definite diagnosis was reached and patient was discharged with fever of unknown etiology as his/her final diagnosis.

### False Positive cases (FP)

The false positive cases included three patients.

One of them had increased uptake in lymph nodes and spleen which was interpreted as malignant in nature. This led to un-necessary lymph node biopsy which showed no pathological malignant process. Final diagnosis of the patients was Infectious mononucleosis according to laboratory results of positive antibodies.

Another one had hyperactive splenomegaly and bone marrow that was interpreted as suspicious for malignancy. Bone marrow biopsy revealed granulocyte.

hyperplasia and hypercellularity with no evidence of ongoing malignancy. The patient was diagnosed clinically and laboratory as Still disease.

The third patient had increased FDG uptake in the nasopharynx and lymph nodes which was reported as suspicious of lymphoma. Nasopharyngeal biopsy was done which was negative for pathological process. The patient had no definite diagnosis upon discharge and on 6 months follow-up with attacks of fever remissions/exacerbation still going.

### False negative cases (FN)

There were two false negative cases in our study, whom only had physiological FDG distribution in their PET/CT examination. The final diagnosis of both of them was urinary tract infection (UTI) which was confirmed by laboratory tests and urine cultures. Both of them had increased Total Leucocytic Count (TLC) and responded well to targeted antibiotic therapy (Table 2).

**Table 2:** Distribution of PET/CT true positive, false positive, true negative and false negative cases among different etiologies of final diagnosis

Final diagnosis	Number of cases	PET/CT positive		PET/CT negative	
	Total: 40	True	False	True	False
Malignant	20	20	0	0	0
Infectious diseases	7	4	1	0	2
NIID	6	5	1	0	0
Fever subsided or no identified diagnosis	7	0	1	6	0

### Diagnostic performance of PET/CT in FUO

The calculated sensitivity and specificity of PET/CT in our study for whole groups of diagnosis were 93.5% and 66.7%, respectively. PET/CT had positive predictive value (PPV) of 90.6% and negative predictive value (NPV) of 75%. Its total calculated diagnostic accuracy was 87.5%.

### Diagnostic value of FDG PET/CT in different disease types

Of the 20 patients with malignant diseases, the detection rate of PET/CT was 100%. FDG PET/CT was able to diagnose four patients out of seven with infectious diseases with detection rate of 57.2%. On the other hand, PET/CT was able to diagnose five patients out of six with non-infectious inflammatory causes as an etiology with detection rate of 83.3%.

Calculated fractionated sensitivity of PET/CT in malignant, infectious and NIID diseases were 100%, 66.6% and 100%, respectively, with the least sensitivity recorded to infectious category.

## Discussion

Fever of unknown origin (FUO) is a diagnostic dilemma that needs integration of various clinical and diagnostic procedures to reach the accurate diagnosis. With the evolution of PET/CT using its combined metabolic and anatomical images, it was possible to diagnose the lesions early compared to other modalities of imaging and diagnosis.

In this study, 18-F-FDG-PET/CT was found to provide useful data, that helped to reach a final clinical diagnosis or ruled out a focal lesion as a cause in patients with FUO. Even a negative PET/CT study was helpful in guiding us for further investigations.

### Malignant causes

The main etiology of FUO in our study was malignant one (50%) and this was different from most of the reported studies in literature where infectious and inflammatory causes predominate. This difference may be caused by the referral bias as this study was done in a tertiary center and inflammatory causes tend to be diagnosed early in the disease process.

In our study, sensitivity of PET/CT to malignant disorders was 100% which matched that reported by most of the previous authors as Singh et al. [9] and Mahajna et al. [10]. Among the malignant causes, lymphoproliferative disorders/hematological malignancies predominated and this was in concordance with the results published by Gafter-Gvili et al. [11] and Zhu et al. [12].

One of the cases in this category was diagnosed with primary bone marrow lymphoma. Primary bone lymphoma (PBL) represents non‐Hodgkin lymphoma (NHL) that primarily arises in the bone marrow without lymphadenopathy. Our patient had multifocal type in the right acetabulum and femur. She had faint easily missed subtle lytic/sclerotic appearance in the CT alone, yet it showed increased FDG uptake in the PET scan which caught the eye and guided the subsequent guided biopsy. As reported by Lim and Ong [13], radiological appearance of PBL can be normal or very subtle, hence, combined anatomical and metabolic imaging plays a major role in the diagnosis.

Another case was diagnosed with solitary plasmacytoma (SP) by pathological examination of left ischial and acetabular hypermetabolic predominantly lytic lesion. Early diagnosis of solitary bone marrow plasmacytoma is particularly important because most of the cases will eventually evolve into multiple myeloma [14]*.*

We also reported a case of Behcet disease that presented with FUO with his PET/CT showing enlarged metabolically active supra and infradiphragmatic lymph nodes which were proven pathologically to be caused by lymphoma. Behcet disease has been reported in the literature to be linked to many types of malignancies Huang et al. [15] and Meydan et al. [16] have reported cases of similar entity for malignant lymphoma complicating cases of Behcet disease.

Other malignant causes of FUO in our study included metastatic colonic cancer to the liver which is a rare established cause in the literature. Liver metastases cause neoplastic fever with many of the patients displaying significant systemic inflammation [17].

Renal cell carcinoma was found in one of our patients which is a reported cause of FUO in the literature [18]*.* Another particularly rare cause of FUO reported in the current study is lung cancer. Non-small cell lung cancer was found by Zee and Soo [19] to present with FUO in diagnosis and relapse, thus being a cause of neoplastic fever.

PET/CT was capable to identify focal sites of early malignancy. In the patient with lung cancer, only small spiculated pulmonary nodule with high PET activity was detected together with metabolically active hilar lymph node in absence of any respiratory symptoms which was then confirmed to be malignant by biopsy.

### Infectious causes

Infectious causes had the lowest detection rate in our study (57.14%) which run in concordance with the studies done before [9, 20]. The two false negative cases in our study belong to this group. It is likely attributed to low sensitivity of PET/CT in lower urinary tract infection (UTI) and the physiological excretion of FDG in urine. This is consistent with the findings reported by Zhu et al. [12] where four of their false negative cases were secondary to UTI.

In addition, we had one false positive case where FDG uptake was located in the nasopharynx, cervical lymph nodes with metabolically active enlarged spleen and diffuse increased bone marrow activity. This was misinterpreted as suspicious for lymphomatous infiltration. The patient refused tissue diagnosis, further investigations revealed positive serology for Infectious Mononucleosis (IMN) and he markedly improved within two months with no recurrence of fever for the following six months. Uptake of FDG in acute Epstein Barre infection has mimicked malignancy in most of the published studies in this subject. For example, Lustberg et al. [21] have reported FDG uptake in cervical and abdominal lymph nodes together with the liver and spleen in their published case report of a patient with acute infectious mononucleosis. Given the ongoing conflict in diagnosis of such cases, we suggest putting Epstein Barre infection as a differential diagnosis in patients presenting with FUO, especially if accompanied with pharyngitis or sore throat having FDG uptake mainly in the cervical lymph nodes and the enlarged spleen. Patients will still need confirmatory serological and/or pathological tests to confirm/rule out the diagnosis.

Two of our infectious cases were secondary to Tuberculous infection. T.B. has been described as the most common infection causing FUO in the non-western countries [3, 22, 23].

As this study was done in the era of COVID-19 pandemic, one of our patients showed COVID-19 pattern of pneumonia as a novel source of infectious FUO. Arita et al. [24] have published a case report in 2021 discussing a similar case with COVID-19 infection presented with prolonged intermittent fever after COVID infection.

### Non-infectious inflammatory diseases

Non-infectious inflammatory diseases were diagnosed in 6 patients in our study with a PET/CT detection rate of 83.3%. One of the six cases was falsely diagnosed by PET/CT as suspicious for lymphoma or leukemia. The patient had increased FDG uptake in the enlarged spleen and diffuse increased uptake in the bone marrow, yet his bone marrow biopsy revealed no evidence of malignancy with only hypercellularity detected. Final diagnosis of the patient by clinical data and laboratory tests was adult-onset Still disease (AOST). Yamashita et al. [25] studied seven AOSD patients who were evaluated by ^18^F-FDG PET/CT. Their PET/CT studies revealed ^18^F-FDG accumulation in the multiple lymph nodes, spleen and BM similar to that observed in malignant lymphoma, which made the differential diagnosis difficult.

Dermatomyositis is a rare cause of FUO in the literature that was found in our study. Lee et al. [26] have reported a similar case presenting as FUO. They concluded that certain subtypes of dermatomyositis can present with atypical presentations as FUO. PET/CT in our case showed diffuse increased FDG uptake in the skeletal muscles mainly muscles of the shoulder girdle, proximal arms, pelvis and thigh muscles.

An interesting finding in our study was that PET/CT was able to successfully diagnose a case of vasculitis affecting the celiac and superior mesenteric arteries. PET/CT has an established role in the literature in diagnosis and monitoring response to therapy in cases with large vessel vasculitis [27]. However, its sensitivity decreases significantly in cases of medium vessel and small vessels vasculitis [28]. In our study, we found increased uptake in the mildly thickened wall of the celiac and superior mesenteric arteries with nearby small metabolically active reactive lymph nodes which are reported findings in cases of vasculitis [29].

### Added value of PET/CT over CT in our study

Beside the benefits of PET as a sensitive modality for lesions with high metabolic activity, benefits of the combined contrast enhanced CT were obtained. CT can assess different chest and abdominal organs in the portal phase of contrast enhancement. In our study, there was a patient with sarcoidosis showing increased mediastinal lymph nodes PET activity. Her lung parenchyma was assessed by CT to clarify the small pulmonary nodules and interstitial pulmonary changes. She had similar findings to that reported in the literature in cases of Sarcoidosis [30]. The lung window interpretation was helpful to rule out the malignant etiology and raise the possibility of inflammatory process.

Among the 40 cases with FUO in our study, PET/CT was able to accurately confirm/exclude the diagnosis in 35 cases with diagnostic accuracy of 87.5%. This is close to the reported accuracy in the previous similar studies as those done by Tokmak et al. [31] and Abdelrahman et al. [32] (90% and 94.5%, respectively).

In our study, the malignant neoplastic lesions showed higher values of standardized uptake values (SUV) in comparison to infectious and NIID lesions. SUV_max_ of infectious and non-infectious inflammatory causes ranged from 3 to 8, while that of the malignant cases ranged from 6 to 45 (Table 1).

### Limitations of PET/CT

Previous studies suggested the high cost of PET/CT as a possible disadvantage. This is not going to be a problem in the very near future because PET/CT scanners are more and more available nowadays. However, its use can be limited to the difficult cases of FUO that the clinical methods and routine investigations fail to determine the underlying cause.

Another concern is the exposure to radiation, which seems to be within acceptable range being similar to the dose the patient receives during other routine radiological examination like whole body CT.

## Conclusions

In conclusion ^18^F-FDG-PET/CT is a recognized tool in the diagnosis and management of FUO. It has a major role in establishing the cause and discriminating between different types of etiologies of FUO. In the future, ^18^F-FDG-PET/CT is expected to be included in the initial diagnostic work-up for the investigation of the etiology of FUO.

## Data Availability

The data and all the material used in this study are available.

## Abbreviations

FUO: Fever of unknown origin
(18F)FDG-PET/CT: Fluorodeoxyglucose positron emission tomography/computed tomography
SUVmax: Maximum standard uptake values
NIID: Non-infectious inflammatory disorders
CT: Computed tomography
TP: True positive
TN: True negative
FP: False positive
FN: False negative
UTI: Urinary tract infections
PBL: Primary bone lymphoma
NHL: Non-Hodgkin Lymphoma
SP: Solitary plasmacytoma
AOST: Adult-onset Still disease
T.B: Tuberculous Bacteria
